# Predicting non-muscle invasive bladder cancer outcomes using artificial intelligence: a systematic review using APPRAISE-AI

**DOI:** 10.1038/s41746-024-01088-7

**Published:** 2024-04-18

**Authors:** Jethro C. C. Kwong, Jeremy Wu, Shamir Malik, Adree Khondker, Naveen Gupta, Nicole Bodnariuc, Krishnateja Narayana, Mikail Malik, Theodorus H. van der Kwast, Alistair E. W. Johnson, Alexandre R. Zlotta, Girish S. Kulkarni

**Affiliations:** 1 Division of Urology, Department of Surgery, University of Toronto, Toronto, ON Canada; 2 Temerty Centre for AI Research and Education in Medicine, University of Toronto, Toronto, ON Canada; 3 Temerty Faculty of Medicine, University of Toronto, Toronto, ON Canada; 4 Georgetown University School of Medicine, Georgetown University, Washington, DC USA; 5 Harvard T.H. Chan School of Public Health, Harvard University, Boston, MA USA; 6 Western University, London, ON Canada; 7 Laboratory Medicine Program, University Health Network, Princess Margaret Cancer Centre, University of Toronto, Toronto, ON Canada; 8 Division of Biostatistics, Dalla Lana School of Public Health, University of Toronto, Toronto, ON Canada; 9 Division of Urology, Department of Surgery, Mount Sinai Hospital, Sinai Health System, Toronto, ON Canada; 10 Division of Urology, Department of Surgery, Princess Margaret Cancer Centre, University Health Network, Toronto, ON Canada

**Keywords:** Bladder cancer, Disease-free survival

## Abstract

Accurate prediction of recurrence and progression in non-muscle invasive bladder cancer (NMIBC) is essential to inform management and eligibility for clinical trials. Despite substantial interest in developing artificial intelligence (AI) applications in NMIBC, their clinical readiness remains unclear. This systematic review aimed to critically appraise AI studies predicting NMIBC outcomes, and to identify common methodological and reporting pitfalls. MEDLINE, EMBASE, Web of Science, and Scopus were searched from inception to February 5th, 2024 for AI studies predicting NMIBC recurrence or progression. APPRAISE-AI was used to assess methodological and reporting quality of these studies. Performance between AI and non-AI approaches included within these studies were compared. A total of 15 studies (five on recurrence, four on progression, and six on both) were included. All studies were retrospective, with a median follow-up of 71 months (IQR 32−93) and median cohort size of 125 (IQR 93−309). Most studies were low quality, with only one classified as high quality. While AI models generally outperformed non-AI approaches with respect to accuracy, c-index, sensitivity, and specificity, this margin of benefit varied with study quality (median absolute performance difference was 10 for low, 22 for moderate, and 4 for high quality studies). Common pitfalls included dataset limitations, heterogeneous outcome definitions, methodological flaws, suboptimal model evaluation, and reproducibility issues. Recommendations to address these challenges are proposed. These findings emphasise the need for collaborative efforts between urological and AI communities paired with rigorous methodologies to develop higher quality models, enabling AI to reach its potential in enhancing NMIBC care.

## Introduction

Non-muscle invasive bladder cancer (NMIBC) has one of the highest per-patient cancer-related costs due to high recurrence rates and need for long-term cystoscopic surveillance^1^. Disease management also profoundly impacts quality-of-life, especially for patients progressing to more advanced disease^2^. Intravesical bacillus Calmette-Guérin (BCG) is the current standard of care for adjuvant treatment in intermediate- and high-risk NMIBC, however up to 40% of patients do not respond to therapy^3^. These “BCG-unresponsive” patients and those who progress from NMIBC to potentially lethal muscle-invasive disease (MIBC) often require aggressive therapy in the form of a radical cystectomy, which carries considerable morbidity and mortality. Therefore, accurate and timely prediction of recurrence and progression remains the cornerstone of management and counselling for NMIBC patients.

Artificial intelligence (AI) has recently emerged as a promising tool in urology, enabling accurate and personalised risk predictions by integrating multimodal data^4^. However, many AI models in urothelial cancer were found to have high risk-of-bias^5^. Indeed, despite the proliferation of AI research, few models have successfully been adopted into clinical practice – underscoring the need for more sophisticated, AI-specific tools to scrutinise these studies. APPRAISE-AI is a quantitative tool we have developed to evaluate both methodological and reporting quality in AI studies^6^. It also provides detailed assessments of data and model quality, making it particularly valuable for comparing AI studies addressing the same clinical question.

This systematic review aims to critically evaluate the robustness of AI models predicting recurrence and progression in NMIBC. We compare the performance of AI and non-AI approaches for these tasks. Using APPRAISE-AI, we assess study quality and identify common methodological and reporting pitfalls. Finally, we provide recommendations to address six key areas: (1) dataset generation, (2) outcome definitions, (3) methodological considerations, (4) model evaluation, (5) reproducibility, and (6) peer-review.

## Results

### Study screening and selection

**Fig. 1:**
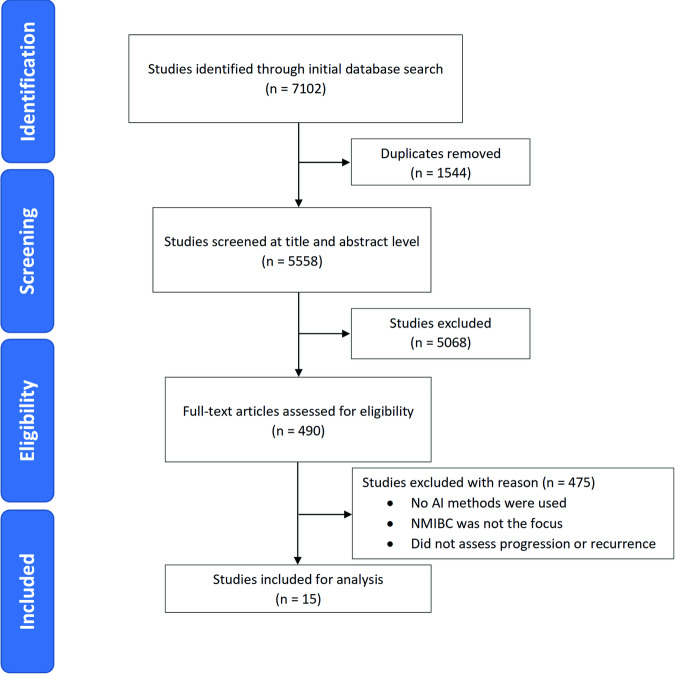
Preferred Reporting Items for Systematic Reviews and Meta-Analyses (PRISMA) flowchart.

The initial search identified 7102 studies, of which 5558 underwent title and abstract screening after removal of duplicates. A total of 490 studies proceeded to full-text review, and 475 were excluded (Fig. 1). In all, 15 studies were included, with five studies focusing on recurrence^7–11^, four on progression^12–15^, and six on both outcomes^16–21^. Detailed characteristics of the included studies are summarised in Tables 1 and 2.

**Table 1:** Study characteristics and performance metrics of studies focused on NMIBC recurrence

Author	Tumour history, NMIBC risk groups, tumour grading scheme	Follow-up duration in months (range, if available)	Definition of recurrence (incidence)	Intravesical therapy	Cohort size	AI model used	Training features (variables)	AI performance (95% CI if available)	Non-AI performance (95% CI if available)
Kim et al.^19^	Primary All risk groups WHO 2004/2016	Median 71	Equivalent or lower pathological stage (28% in validation)	BCG	Train: 103 Test: 32	Deep belief network	Genetic profile of NMIBC subtypes (ex. DP.BCG + , REC.BCG + , EP)	Acc: 89	EORTC: Acc: 56 Sens: 65 Spec: 47 AUC: 0.57
Xu et al.^9^	Primary & recurrent All risk groups WHO 2004/2016	NR	Any stage within the first 2 years (50% in training, 43% in validation)	NR	Train: 50 Test: 21	SVM, LASSO	Age, sex, grade, muscle-invasive status, and 32 radiomics features based on tumour regions of interest	SVM: Acc: 82 Sens: 84 Spec: 80 AUC: 0.86 (0.84−0.88) LASSO: Acc: 72 Sens: 74 Spec: 75 AUC: 0.75 (0.74−0.70)	NR
Caiet et al.^8^	Recurrent High-risk only WHO 1973	Mean 108 (91−149)	Any stage or papillary formations seen on cystoscopy (62%)	BCG, MMC	Train: 100 Test: 43	ANN	Sex, age, stage, grade, previous recurrence rate, response to BCG therapy, number of tumours, tumour size, presence of tumour-associated inflammatory reaction, adjuvant therapy post-TURBT	Acc: 84 Sens: 82 Spec: 96 AUC: 0.82 PPV: 98 NPV: 92	NR
Ji^7^	NR	NR	NR (55%)	NR	Train: 321 (Test on same cohort)	Radial basis function network	Histology, grade, lymph nodes, bilharziasis history, stage, DNA ploidy, sex, age interval	Acc: 91 Sens: 60 Spec: 68	NR
Jobczyk et al.^21^	Primary All risk groups WHO 1973	Median 13 (0.04−132)	High grade, T1, or CIS (45% in training, 63% in validation)	BCG, MMC	Train: 3570 Test: 322	DeepSurv	Sex, age, stage, grading, number of tumours, tumour size, EORTC and CUETO scores, and type of intravesical treatment	AUC: 0.66 (0.66−0.67)	EORTC: AUC: 0.64 (0.61−0.68)
Lucas et al.^11^	Primary & recurrent All risk groups WHO 1973	NR	Any stage (27% at 1-yr follow-up, 63% at 5-yr follow-up)	BCG, MMC	At 1-yr: Train: 215 Validation: 72 Test: 72 At 5-yrs: Train: 169 Validation: 56 Test: 56	VGG16	Digitised formalin-fixed paraffin-embedded histopathology slides combined with patient, tumour, and treatment characteristics	5-yr follow-up: Acc: 74 (60−85) Sens: 89 (71−98) Spec: 57 (34−77) AUC: 0.76 (0.62−0.87)	Logistic regression for 5-yr follow-up: Acc: 52 (37−66) Sens: 67 (46−83) Spec: 35 (16−57) AUC: 0.57 (0.41−0.73)
Lee et al.^20^	Primary All risk groups WHO 2004/2016	Median 36 (7−70)	Ta, T1, or CIS (61%)	BCG	Train: 122 (Test on same cohort)	SVM	Age, smoking history, urine cytology, prostate volume, intravesical prostatic protrusion, stage, grade, tumour size, number of tumours, CIS, BCG	Acc: 80 AUC: 0.75 F1: 0.80	NR
Qureshi ^16^	Primary Unspecified risk groups WHO 1973	Mean 27 (1−96)	Equivalent or lower pathological stage within 6 months (50% in training, 50% in validation)	NR	Train: 36 Test: 20	ANN	Stage, grade, tumour size, number of tumours, sex, EGFR status, smoking history, histology, CIS, tumour metaplasia, tumour architecture, tumour site, c-erbB2 and p53 status	Acc: 75 Sens: 70 Spec: 80	Consultant urologists: Acc: 79 Sens: 75 Spec: 83
Fujikawa et al.^17^	Primary All risk groups WHO 1973	Mean 192 (180−233)	Equivalent or lower pathological stage (47%)	BCG, MMC	Train: 68 Testing: 22	ANN	Stage, grade, number of tumours, age, sex, tumour architecture, estimates of mean nuclear volume	Sens: 33 Spec: 40 PPV: 33 NPV: 40	NR
Lopez de Maturana et al.^18^	Primary All risk groups WHO 2004/2016	NR	Ta, T1, or CIS (33%)	NR	Train: 822 Test: 10-fold cross validation	Bayesian and LASSO regression	Area, sex, number of tumours, stage, grade, tumour size, treatment, SNPs	AUC: 0.61	NR
Tokuyama et al.^10^	Primary All risk groups WHO 2004/2016	Median 73 (24−192)	Any stage (35% in training, 40% in validation)	Induction BCG, MMC	Train: 95 Test: 30	SVM, RF	960 morphologic features from each tumour region of interest	SVM: Acc: 90 Sens: 100 Spec: 83 RF: Acc: 87 Sens: 100 Spec: 78	NR

*Acc* accuracy, *ANN* artificial neural network, *AUC* area under the curve, *BCG* Bacillus Calmette-Guerin, *CUETO* Club Urológico Español de Tratamiento Oncológico, *EORTC* European Organisation for Research and Treatment of Cancer, *LASSO* least absolute shrinkage and selection operator, *MMC* mitomycin C, *NR* not reported, *RF* random forest, *Sens* sensitivity, *Spec* specificity, *SVM* support vector machine.

**Table 2:** Study characteristics and performance metrics of studies focused on NMIBC progression

Author	Tumour history, NMIBC risk groups, tumour grading scheme	Follow-up duration in months (range, if available)	Definition of progression (incidence)	Intravesical therapy	Cohort size	AI model used	Training features (variables)	AI performance (95% CI if available)	Non-AI performance (95% CI if available)
Kim et al.^19^	Primary All risk groups WHO 2004/2016	Median 71	≥ T2 (25% in validation)	BCG	Train: 103 Test: 32	Deep belief network	Genetic profile of NMIBC subtypes (ex. DP.BCG + , REC.BCG + , EP)	Acc: 75	EORTC: Acc: 34 Sens: 0 Spec: 88 AUC: 0.53
Abbod et al.^12^	Recurrent All risk groups WHO 1973	Median 36	From Ta to T1 (50%)	NR	Train: 67 (Test on same cohort)	ANN, NFM	Gene expression profiles from tissue microarray analysis of non-invasive and invasive bladder cancer	ANN: Acc: 100 RMS: 5.18 NFM: Acc: 100 RMS: 2.2	Logistic regression: RMS: 13.2
Jobczyk et al.^21^	Primary All risk groups WHO 1973	Median 13 (0.04-132)	Increase to T1, T2, N + , M + , or low to high grade (8% in training, 12% in validation)	BCG, MMC	Train: 3570 Test: 322	DeepSurv	Sex, age, stage, grading, number of tumours, tumour size EORTC and CUETO scores, and type of intravesical treatment	AUC: 0.88 (0.87-0.88)	EORTC: AUC: 0.82 (0.77-0.86)
Lee et al.^20^	Primary All risk groups WHO 2004/2016	Median 36 (7-70)	≥ T2 or M+ (9%)	BCG	Train: 122 (Test on same cohort)	SVM	Age, smoking history, urine cytology, prostate volume, intravesical prostatic protrusion, stage, grade, tumour size, number of tumours, CIS, BCG	Acc: 80 AUC: 0.75 F1: 0.80	NR
Qureshi ^16^	Primary Unspecified risk groups WHO 1973	Mean 27 (1-96)	From Ta to T1 or T1 to T2 (16% in training, 17% in validation)	NR	Train: 45 Test: 60	ANN	Stage, grade, tumour size, number of tumours, EGFR status	Acc: 80 Sens: 70 Spec: 82	Consultant urologists: Acc: 74 Sens: 55 Spec: 78
Catto ^13^	Primary All risk groups WHO 1973	Median 96 (1-204)	Higher stage or grade (NR)	BCG, MMC	Train: 64 Validation: 32 Test: 11	NFM, ANN	Stage, grade, age, sex, smoking status, immunohistochemical expression of p53, methylation of 11 loci	NFM: Acc: 100 Sens: 100 Spec: 100 AUC: 1 ANN: Acc: 99 Sens: 97 Spec: 100 AUC: 1	Logistic regression: Acc: 74 Sens: 65 Spec: 80 AUC: 0.86
Fujikawa et al.^17^	Primary All risk groups WHO 1973	Mean 192 (180-233)	≥ T2 (19%)	BCG, MMC	Train: 68 Test: 22	ANN	Stage, grade, number of tumours, age, sex, tumour architecture, estimates of mean nuclear volume	Sens: 100 Spec: 67 PPV: 40 NPV: 100	NR
Lopez de Maturana et al.^18^	Primary All risk groups WHO 2004/2016	NR	≥ T2, M + , or bladder cancer death (9%)	NR	Train: 810 Test: 10-fold cross validation	Bayesian and LASSO regression	Area, sex, number of tumours, stage, grade, tumour size, treatment, SNPs	AUC: 0.76	NR
Yates et al.^14^	Primary All risk groups WHO 1973	Median 24	Higher stage or grade (33%)	NR	Train: 57 Validation: 29 Test: 10	NFM	Methylation frequencies of 17 gene promoters	Acc: 90 Sens: 75 Spec: 97	Cox regression: Sens: 97 Spec: 38 AUC: 0.67
Catto et al.^15^	Primary & recurrent All risk groups WHO 1973	Median 89 (2-154)	From Ta to T1 or T1 to T2 (20%)	NR	Train: 178 Validation: 89 Test: 29	NFM, ANN	Among panel of 200 progression-related genes, 11 of the highest-ranked genes were chosen	AUC: 0.66	NR

*Acc* accuracy, *ANN* artificial neural network, *AUC* area under the curve, *BCG* Bacillus Calmette-Guerin, *CUETO* Club Urológico Español de Tratamiento Oncológico, *EORTC* European Organisation for Research and Treatment of Cancer, *LASSO* least absolute shrinkage and selection operator, *MMC* mitomycin C, *NR* not reported, *RF* random forest, *Sens* sensitivity, *Spec* specificity, *SVM* support vector machine.

### Study characteristics

Seven studies (47%) were published between 2015 and 2022, while eight (53%) were published between 2000 and 2010. Most studies (60%) were from Europe (five from United Kingdom, one from each of Spain, Poland, Netherlands, and Italy), followed by Asia (two from each of Japan and South Korea, one from China) and Africa (one from Egypt).

All studies focused on model development using retrospective data, of which four (27%) included multiple institutions. Only one study included non-academic institutions^18^. Median sample size was 125 (IQR 93−309) and median follow-up was 71 months (IQR 32−93). Median recurrence and progression rates were 50% (IQR 42−62) and 19% (IQR 12−25), respectively.

### Patient characteristics

Most studies included all NMIBC risk groups. However, patients varied with respect to prior NMIBC history, with nine studies (60%) including only primary tumours, two (13%) with exclusively recurrent tumours, three (20%) with both, and one (7%) with no details provided. Tumour grading scheme also varied, with nine studies (60%) using the WHO 1973 classification system, five (33%) using WHO 2004/2016, and one (7%) with no details provided. Four studies (27%) explicitly reported use of repeat transurethral resection of bladder tumour (TURBT)^10,11,20,21^. Eight studies (53%) mentioned administration of intravesical therapy, of which six used both BCG and mitomycin C while two used only BCG.

### Outcome definitions

Various definitions of recurrence and progression were described. Seven definitions were used for recurrence, including relapse of: (1) equivalent or lower stage, (2) equivalent or lower stage within six months, (3) any stage, (4) any stage within two years, (5) any stage or papillary formations on cystoscopy, (6) Ta, T1, or CIS, and (7) high-grade, T1, or CIS. For progression, seven definitions were reported, including relapse of: (1) ≥ T2, (2) ≥ T2 or metastases, (3) ≥ T2, metastases, or bladder cancer death, (4) from Ta to T1, (5) from Ta to T1 or T1 to T2, (6) from Ta/CIS to T1, T2, nodal disease, metastases, or from low to high grade, and (7) higher stage or grade.

### Model characteristics

The most commonly used AI models were based on neural networks (*n* = 11, 73%), including shallow neural networks, neuro-fuzzy modelling, deep belief networks, DeepSurv, and convolutional neural networks. Studies differed in how their models were trained and evaluated, with seven studies (47%) using separate training and testing cohorts; four (27%) using separate training, validation, and testing cohorts; one (7%) performing 10-fold cross-validation; and three (20%) using the same cohort for both training and testing. Most models incorporated clinicopathological features (*n* = 10), while other data types included gene expression profiles (*n* = 6) and radiomic features (*n* = 2).

Median c-index was 0.76 (IQR 0.68−0.81) for recurrence and 0.76 (IQR 0.75−0.88) for progression. Three studies (20%) provided calibration plots to assess reliability of risk estimates and only one assessed net benefit using decision curve analysis.

### Quality of studies

Interrater reliability of APPRAISE-AI was moderate to excellent, with ICCs ranging from 0.60−1 for item scores, 0.83−0.96 for domain scores, and 0.98 for overall scores (Supplementary Table 1). Median overall score was 37 (low quality) and ranged from 26 (low quality) to 64 (high quality). From 2000 to 2010, all studies were low quality, except for one moderate quality (Supplementary Fig. 1). From 2010 to 2022, three of seven studies were low quality. Overall study quality improved over time (regression coefficient 0.65, 95% CI 0.08−1.21, *p* = 0.03). Only one study throughout the entire study period was high quality^21^.

**Fig. 2:**
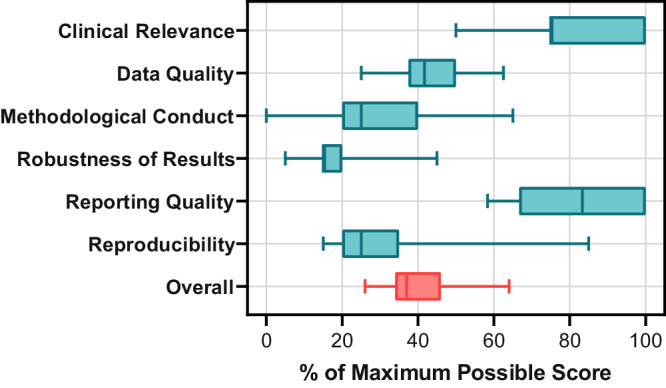
APPRAISE-AI domain and overall scores. Box plot of APPRAISE-AI domain (blue) and overall (red) scores for the 15 studies using AI to predict NMIBC recurrence and progression. Each box represents the 25th and 75th percentiles with the centre line indicating the median, and the whiskers extending to the minimum and maximum scores. Each field is presented as a percentage of the maximum possible score for that field (i.e., consensus score/maximum possible score x 100%) to compare scores between fields, irrespective of the assigned weighting. Overall APPRAISE-AI scores were graded as follows: very low quality, 0-19; low quality, 20−39; moderate quality, 40−59; high quality, 60−79; very high quality, 80−100.

The two strongest APPRAISE-AI domains were clinical relevance and reporting quality, while the three weakest were methodological conduct, robustness of results, and reproducibility (Fig. 2). Items achieving greater than 60% of their maximum possible score included title, background, objective and problem, eligibility criteria, ground truth (defining outcome of interest), model description, cohort characteristics, model specification, critical analysis, implementation into clinical practice, and disclosures (Supplementary Fig. 2). Items achieving less than 40% of their maximum possible score included source of data, data abstraction, cleaning, and preparation, sample size calculation, baseline, hyperparameter tuning (adjusting attributes to influence how models learns from data), clinical utility assessment, bias assessment, error analysis, and transparency. Three studies described how missing data were handled, of which one used complete-case analysis and two imputed missing values using random forests. No studies reported on sample size calculation. Only one study included a publicly accessible repository containing the data and AI models necessary to replicate their findings^21^.

### Comparison between AI and non-AI approaches

**Fig. 3:**
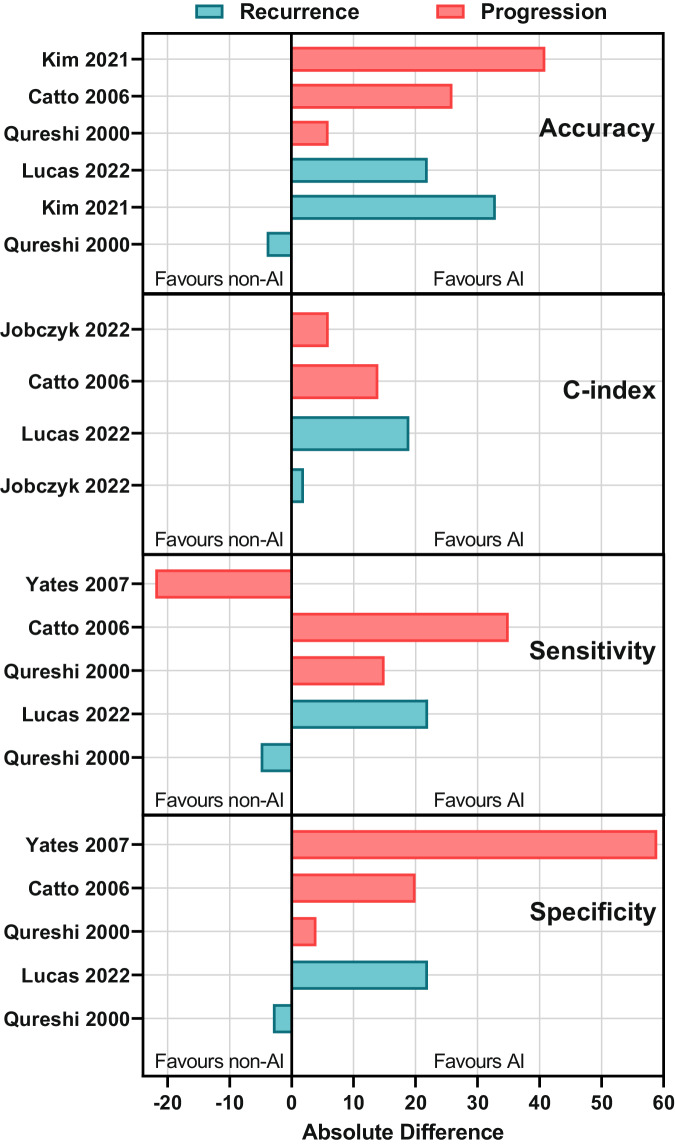
Differences in performance between AI and non-AI approaches. Absolute difference in reported performance metrics between AI and non-AI approaches, stratified by recurrence or progression prediction task.

Seven studies (47%) compared AI models with non-AI approaches. These included regression-based models (logistic or Cox regression, *n* = 4), existing nomograms (European Organisation for Research and Treatment of Cancer nomogram, *n* = 2), and clinical experts (*n* = 1). Most studies found that AI outperformed non-AI methods for both recurrence and progression (Fig. 3). However, two studies, which compared AI versus urologists and Cox regression, found that non-AI approaches were superior for some metrics. The margin of benefit of AI compared to non-AI approaches varied depending on study quality. Median absolute difference in performance between AI and non-AI approaches was 10 for the ten low quality studies, 22 for the four moderate quality studies, and 4 for the one high quality study (Supplementary Fig. 3).

## Discussion

This systematic review identified 15 studies predicting NMIBC recurrence and progression. A distinguishing feature is the use of APPRAISE-AI to provide a comprehensive summary of the methodological rigour and reporting quality of these studies. While most studies reported good to excellent performance of their AI models, two-thirds were rated as low quality. Only one study in the last two decades was considered high quality^21^. Although the clinical relevance and reporting quality domains attained the highest scores, methodological conduct, robustness of results, and reproducibility consistently ranked the lowest – a recurring issue among other clinical AI studies^5,22^. This discrepancy between high reporting quality yet poor reproducibility can be explained by the former domain encompassing familiar elements such as cohort characteristics, critical analysis, limitations, and disclosures. These items are well understood and routinely reported by the medical community, and often mandated by journals. In contrast, the reproducibility domain introduces AI-specific concepts including model description, hyperparameter tuning, model specification, and data/model transparency. These items, unique to AI studies, may not be comprehensively addressed within current reporting practices. Therefore, this review emphasises the need for better methodological and reporting practices tailored for AI studies within urology^23–25^.

### Common pitfalls of current studies

Common pitfalls can be categorised into dataset limitations, heterogeneous outcome definitions, methodological flaws, inadequate model evaluation, and reproducibility issues. These concerns may lead to overly optimistic estimates of model performance and limit their potential for clinical use.

**Datasets**: Most models were trained on retrospective cohorts from single academic institutions, thus may lack generalisability in non-academic settings, such as community hospitals. Median cohort size was 125, which is considered small even for regression-based methods. Models trained on smaller datasets are at risk of *instability*, defined as volatility in models and their predictions because of their dependence on the training data and modelling approaches used^26^. Unstable models may generate unreliable predictions, especially when applied to external cohorts.

Data quality issues were also attributed to substantial heterogeneity in eligibility criteria, patient, and tumour characteristics. Only 20% of models were trained on both primary and recurrent tumours. Studies were also divided in their use of the WHO 1973 or 2004/2016 grading schemes. In addition, standard of care varied – only 27 and 53% of studies reported using repeat TURBT and intravesical therapies, respectively, despite almost all studies including high-risk patients for whom these treatments would be recommended. These findings highlight the need for diverse, representative data that accurately reflects the NMIBC patient population and current standard of care^27,28^.

**Outcome definitions**: Despite focusing this review on only two prediction tasks (recurrence and progression), we identified 14 distinct definitions across 15 studies for these outcomes. These variations in outcome definitions substantially limit comparability of studies.

**Methods**: Methodological errors were frequently repeated in studies. There was limited clarity on data pre-processing steps, especially regarding handling of missing data. Similarly, hyperparameter tuning steps, which defines how models learns from data, were poorly described. In addition, no sample size calculations were reported, thus it is unclear whether there were sufficient events per predictor variable for model training^29^. These concerns undermine transparency of datasets and models.

Several studies had concerns for data leakage – for example, using the same dataset for model training and testing without additional steps to obtain an optimism-corrected estimate of model performance^30^. Indeed, we found that studies with data leakage reported a median accuracy of 86% (IQR 80−93) compared to 83% (IQR 76−90) for those without this concern. Over half of studies (8/15) did not compare their AI models with alternative approaches, such as existing nomograms, statistical models, or clinical judgement. Of the remaining that provided a comparison, we found that better study quality was associated with a lower margin of benefit of AI models.

**Evaluation**: Studies typically reported on accuracy, sensitivity, specificity, and c-index. However, these measures are not always appropriate. Furthermore, measures of statistical significance for performance metrics, calibration plots, and net benefit were rarely reported. Therefore, researchers are encouraged to understand the strengths and limitations of different evaluation metrics to select the most relevant ones for addressing their clinical question^31–33^.

Algorithmic bias refers to disparities in AI performance for clinically relevant subgroups, such as sex, race, and socioeconomic status – which violates the ethical principle of justice^28^. These inequities underscore the fundamental link between training data and model behaviour. Non-representative data may introduce biases against minority groups, which in turn may perpetuate discriminatory practices within AI models. Indeed, several studies have found that AI models disproportionately affect marginalised patients, including females, individuals of African ancestry, and lower socioeconomic status^34,35^. Various strategies have been proposed to mitigate algorithmic bias to develop “fair” AI models. For instance, a bias assessment is recommended for examining performance heterogeneity across clinically relevant subgroups, similar to subgroup analyses commonly reported in clinical trials^6,23,28,36^. However, only two studies conducted some form of bias assessment, highlighting a gap in current evaluation practices.

**Reproducibility**: Only one study provided publicly accessible datasets and code necessary to replicate their findings. This so-called “reproducibility crisis” is concerning and consistent with other areas of AI in medicine^37^. Since clinical AI models often involve high-stakes decisions with direct patient consequences, failure to reproduce study findings may erode trust in these models and lead to poor clinical adoption.

### Recommendations

Despite notable improvements in study quality, substantial work remains to address common pitfalls outlined in this review. We provide the following recommendations to enhance quality of future AI studies in NMIBC, which are summarised in Table 3.

**Table 3:** Summary of recommendations to improve AI studies in NMIBC prognostication

Areas for improvement	Recommendations
Data quality	1. Include all types of NMIBC patients, regardless of stage, grade, tumour history, subtype, or divergent differentiation 2. Include patients treated at non-academic institutions 3. Included patients should reflect standard of care (i.e., repeat TURBT, intravesical BCG) 4. Include both WHO 1973 and WHO 2004/2022 tumour grading schemes, where possible 5. Ensure adequate sample size is available prior to model development
Outcome definitions	1. Adopt definitions outlined by the International Bladder Cancer Group^41^ Recurrence: relapse of any stage or grade, development of muscle-invasive, nodal, or metastatic disease Grade progression: transition from low to high grade disease Stage progression: transition from Ta or CIS to T1 disease, development of muscle-invasive, nodal, or metastatic disease
Methodology	1. Clearly describe data pre-processing, model development, and hyperparameter tuning steps 2. Isolate the testing cohort prior to any data pre-processing or model training to prevent data leakage 3. Incorporate methods to address model overfitting (i.e., bootstrapping, internal cross-validation, or external validation)
Evaluation	1. Compare AI model(s) with established clinical prediction models (i.e., EAU NMIBC risk calculator), other published models, and/or regression-based approaches 2. Evaluate AI model(s) based on discrimination, calibration, net benefit, and bias
Reproducibility	1. Share models, code, and data in public repositories (i.e., GitHub)
Reviewers	1. Recruit reviewers with AI expertise to evaluate technical aspects of AI studies 2. Assess studies based on data quality, outcome definitions, methodological conduct, robustness of results, and reproducibility

*AI* artificial intelligence, *BCG* bacillus Calmette-Guérin, *CIS* carcinoma-in-situ, *EAU* European Association of Urology, *NMIBC* non-muscle invasive bladder cancer, *TURBT* transurethral resection of bladder tumour, *WHO* World Health Organisation.

**Recommendations for data quality**: Datasets should be inclusive of NMIBC patients, regardless of their tumour history, stage, grade, subtype, or divergent differentiation, and should not be restricted to academic institutions. Study cohorts should also reflect standard of care, including use of repeat TURBT and intravesical therapies. For example, the European Association of Urology (EAU) prognostic risk groups were based on primary NMIBC patients who did not receive intravesical BCG^38^. Consequently, these risks groups were found to overestimate progression risk in contemporary BCG-treated patients^39^. As there is no international consensus on NMIBC grading, researchers are encouraged to report both WHO 1973 and 2004/2022 grading whenever feasible. This topic remains controversial, although proponents have advocated for a hybrid grading system^40^.

Adequate sample size is also essential to ensure model stability. A sample size calculation example is provided in Supplementary Note 2.

**Recommendations for outcome definitions**: To enhance consistency, researchers are encouraged to refer to definitions outlined by the International Bladder Cancer Group^41^. Additional patient-centred outcomes include number of invasive procedures administered over a two-year timeframe and need for cystectomy, radiation, or systemic chemotherapy^42^.

**Recommendations for methodology**: Researchers are encouraged to refer to relevant AI reporting guidelines from the Enhancing the QUAlity and Transparency Of health Research (EQUATOR) Network based on their data types and study context (i.e., model development, validation, or clinical trials). For example, the Standardised Reporting of Machine Learning Applications in Urology (STREAM-URO) framework outlines best practices in reporting AI studies in urology^23^. These include describing: (1) how datasets were divided into training and testing cohorts, (2) how data were pre-processed or modified, (3) how missing data were handled, and (4) what hyperparameters were tuned and how (i.e., grid search, optimisation metric). To prevent data leakage, it is imperative to isolate the testing cohort prior to any data pre-processing steps such as normalisation or imputation. Studies should also incorporate methods to address model overfitting, such as bootstrapping, internal cross-validation, or external validation^33^.

**Recommendations for evaluation**: Researchers are recommended to compare AI models with appropriate baselines such as previously published models or regression-based approaches. These comparators help justify whether additional complexity and opacity of AI approaches are warranted. Model evaluation should encompass measures of discrimination (c-index), calibration (calibration plot), and net benefit (decision curve analysis). Furthermore, we advocate for the use of bias assessments to assess for performance heterogeneity across clinically relevant subgroups, such as age group, sex, and ethnicity.

**Recommendations for reproducibility**: We recognise that institutional privacy and intellectual property considerations may impose restrictions on data and code sharing. However, researchers are strongly encouraged to disseminate their models via publicly accessible platforms or web applications. This practice is best exemplified by Jobczyk et al., who provided a web application for their model and made their deidentified datasets and code available in a public repository^21^. Alternatively, data can be securely housed in dedicated environments designed for clinical information, as done for electronic health record data from the Beth Israel Deaconess Medical Center in the Medical Information Mart for Intensive Care^43^.

**Recommendations for reviewers**: In line with current journal practices of including statistical reviewers, editorial boards may consider recruiting reviewers with AI expertise to assess technical aspects of these studies. Furthermore, we recommend reviewers pay close attention to common pitfalls identified in this review, including methodological conduct, robustness of results, and reproducibility. APPRAISE-AI may be valuable in providing an overall assessment of study quality and identifying specific concerns that may be clarified with study authors^6^.

### Bridging the gap in the adoption of AI reporting guidelines

Despite the proliferation of AI reporting guidelines in recent years, the methodological and reporting pitfalls outlined in this review were consistent with those identified in other areas of medicine, including medical imaging^44–46^, ophthalmology^47^, vascular surgery^48^, neurosurgery^49^, and oncology^50,51^. One possible explanation may be due to a translational gap between guideline developers and other researchers conducting AI studies. For instance, Pattathil et al. reviewed randomised controlled trials evaluating AI interventions in ophthalmology based on adherence to the CONSORT-AI checklist, a reporting guideline for AI clinical trials^47,52^. Although three trials were published following the release of CONSORT-AI, guideline adherence ranged from 37 to 78%. However, none of the trial investigators were involved in the development of this guideline. We recently evaluated AI studies on paediatric hydronephrosis using STREAM-URO and APPRAISE-AI^53^. Among the three studies published after the introduction of these frameworks, the highest scoring study was authored by the same group that developed these tools. These findings reinforce the need for broader stakeholder engagement during guideline development, stronger collaborations between the medical and AI communities, and most importantly, mandating the use of appropriate AI reporting guidelines by journals. Recent initiatives, such as the TRIPOD-AI (prediction models)^54^, PRISMA-AI (systematic reviews and meta-analyses)^55^, and CANGARU guidelines (generative AI and large language models)^56^, are notable examples that prioritise these considerations.

### Data and practice variation due to the human nature of medicine

Despite best practices in AI, the inherent human nature of medicine may impact model generalisability. Tumour staging and grading – which are fundamental in NMIBC prognostication – are subject to considerable interobserver and intraobserver variability, with kappa scores ranging from 0.42 to 0.60 for staging, 0.003−0.68 for the WHO 1973 grading system, and 0.17−0.70 for the WHO 2004/2016 grading system^57,58^. Furthermore, the RESECT study has highlighted significant variability in recurrence rates among institutions even after controlling for known risk factors, suggesting that differences in surgical technique and perioperative management may play a role^59^. These limitations require additional efforts to minimise practice variation to allow AI to achieve its full potential.

### Limitations

Our findings should be interpreted within the context of its limitations. Importantly, study quality was determined using APPRAISE-AI, which was published following the studies included in this review. Accordingly, best practices in AI may have evolved over time. Nevertheless, APPRAISE-AI is well-aligned with established non-AI reporting guidelines such as the Transparent Reporting of a multivariable prediction model for Individual Prognosis Or Diagnosis (TRIPOD) statement^60^. Therefore, improved adherence to these guidelines may be reflected in better APPRAISE-AI scores in recent years. In addition, performance metrics could not be pooled across studies due to inconsistent reporting of these metrics and confidence intervals. Therefore, a more sophisticated comparison between AI and non-AI approaches could not be conducted. Finally, only 15 studies were included given the focused scope of this review. However, we also incorporated studies from non-clinical journals, such as those found in the Institute of Electrical and Electronics Engineers (IEEE) family of publications.

In conclusion, this systematic review examined current AI applications to predict recurrence and progression in NMIBC. Despite some progress in study quality, majority of studies were deemed low quality and likely unsuitable for clinical use. Common pitfalls revolved around dataset limitations, heterogeneous outcome definitions, methodological flaws, suboptimal model evaluation, and reproducibility concerns, notwithstanding limitations due to variability in pathological assessment, surgical technique, and perioperative management. Specific recommendations are provided for researchers and reviewers to ensure best practices in AI are followed. Key stakeholders should prioritise enhancing dataset curation, refining methodological approaches, and improving transparency and completeness of reporting. These concerted efforts are vital in developing high quality AI models that can safely be integrated into future NMIBC care.

## Methods

This systematic review was conducted in accordance with the Preferred Reporting Items for Systematic Reviews and Meta-analyses (PRISMA) guidelines and was prospectively registered on PROSPERO (CRD42022354048). There were no deviations from the PROSPERO analytical plan.

### Search strategy

OVID MEDLINE, EMBASE, Web of Science, and Scopus were searched from inception to February 5th, 2024. The search strategy was based on a recent scoping review on AI applications in urothelial cancer, including both bladder cancer and upper tract urothelial carcinoma (search strategy available in Supplementary Note 1)^5^.

### Eligibility criteria

All studies investigating the use of AI to predict recurrence or progression in patients with pathologically confirmed NMIBC were included. AI was defined as the use of a computer system to mimic human cognitive functions for clinical decision support. AI models included tree-based models, support vector machines, artificial neural networks, deep learning, and natural language processing. Recurrence was defined as the first relapse of bladder tumour (any stage) following initial diagnosis of NMIBC, or as defined by study investigators. Progression was defined as the first relapse of bladder tumour invading the muscularis propria (T2) following initial diagnosis of NMIBC, or as defined by study investigators. Only studies written in English were included.

Studies were excluded if AI approaches were not used, or non-bladder cancer neoplasms were described. Studies were also excluded if the primary aim was to detect T2 disease on imaging (i.e., diagnostic study) or to assess risk factors rather than prediction modelling. Reviews, abstracts, and articles without full text were excluded.

### Data extraction and synthesis

Four reviewers (JW, SM, NB, KN) independently screened and abstracted eligible studies, with disagreements resolved by consensus. The following data were collected: study demographics, patient and tumour characteristics, definition of recurrence and progression, sample size, types of AI models, training features, performance metrics, and information relevant to the evaluation of study quality.

### Quality assessment using APPRAISE-AI

APPRAISE-AI is a scoring tool designed to evaluate methodological and reporting quality of AI studies for clinical decision support^6^. Articles were scored using a standardised form consisting of 24 items with a maximum overall score of 100 points. Each APPRAISE-AI item was mapped to one of six domains: clinical relevance, data quality, methodological conduct, robustness of results, reporting quality, and reproducibility. Overall scores were interpreted as follows: 0−19, very low quality; 20−39, low quality; 40−59, moderate quality; 60−79, high quality; and 80−100, very high quality. Collectively, the APPRAISE-AI item, domain, and overall scores provide macro- and micro-level insights on the strengths and weaknesses of each study.

Two reviewers (JCCK, AK) experienced in developing urological AI applications independently evaluated each article. Disagreements were resolved by a re-review of the article, APPRAISE-AI item criteria, and discussion until a consensus was reached. Interrater reliability was measured using intraclass correlation coefficients (ICCs; calculated with two-way random effects, absolute agreement, and single measurement). ICC values less than 0.50 indicated poor reliability, values between 0.50 and 0.75 indicated moderate reliability, values between 0.75 and 0.90 indicated good reliability, and values greater than 0.90 indicated excellent reliability^61^. Linear regression was used to determine whether overall APPRAISE-AI scores improved over time.

### Comparison between AI and non-AI approaches

Performance was compared between AI and non-AI approaches examined within the included studies. Non-AI models included statistical models, clinical judgement, or existing clinical tools, such as the European Organisation for Research and Treatment of Cancer nomogram^62^. Accuracy, c-index, sensitivity and specificity were considered for this analysis since these metrics were most commonly reported. If studies reported metrics for multiple cohorts, we selected metrics based on the following hierarchy: external validation, internal validation, and training cohort. For each study, the absolute performance difference between the best AI and non-AI model was recorded separately for recurrence and progression. All analyses were performed using GraphPad PRISM version 8.3.0 and MedCalc version 19.6.3.

### Supplementary information

Supplementary Material

Dataset

## Data Availability

A public Github repository (10.5281/zenodo.7930888) has been established for researchers to use the APPRAISE-AI tool. The source data for all figures are included as a Supplementary Data file.
