# Supplementary material for: Predicting non-muscle invasive bladder cancer outcomes using artificial intelligence: a systematic review using APPRAISE-AI
Source: NPJ Digit Med. 2024 Apr 18;7:98. doi: 10.1038/s41746-024-01088-7 (PMC11026453; doi:10.1038/s41746-024-01088-7)
Supplement: Supplementary file 1 — Supplementary Material [file 41746_2024_1088_MOESM1_ESM.pdf]

Table of Contents

Supplementary Note 1. Search strategy.................... 2
Supplementary Table 1.................... 5
Supplementary Figure 1.................... 6
Supplementary Figure 2.................... 7
Supplementary Figure 3.................... 8
Supplementary Note 2. Sample size calculation.................... 9
Supplementary References.................... 10

Supplementary Note 1. Search strategy

MEDLINE

1. exp Artificial Intelligence/
2. exp Diagnosis, Computer-Assisted/
3. exp Pattern Recognition, Automated/
4. exp Image Processing, Computer-Assisted/
5. exp Machine Learning/
6. exp Deep Learning/
7. exp Natural Language Processing/
8. artificial intelligen*.tw,kf.
9. machine learn*.tw,kf.
10. neural network*.tw,kf.
11. deep learn*.tw,kf.
12. computer vision.tw,kf.
13. natural language process*.tw,kf.
14. 1 or 2 or 3 or 4 or 5 or 6 or 7 or 8 or 9 or 10 or 11 or 12 or 13
15. exp Carcinoma, Transitional Cell/
16. exp Urinary Bladder Neoplasms/
17. exp Ureteral Neoplasms/
18. exp Urethral Neoplasms/
19. exp Kidney Neoplasms/
20. ((urothelial or transitional cell* or transition cell*) adj3 (carcinoma or neoplasm or cancer)).tw,kf.
21. (bladder adj3 (carcinoma or neoplasm or cancer)).tw,kf.
22. (ureteral adj3 (carcinoma or neoplasm or cancer)).tw,kf.
23. (urethral adj3 (carcinoma or neoplasm or cancer)).tw,kf.
24. ((renal pelvis or ureteropelvic junction) adj3 (carcinoma or neoplasm or cancer)).tw,kf.
25. 15 or 16 or 17 or 18 or 19 or 20 or 21 or 22 or 23 or 24
26. 14 and 25

Embase

1. exp artificial intelligence/
2. exp machine learning/
3. exp deep learning/
4. exp natural language processing/
5. artificial intelligen*.tw,kf.
6. neural network*.tw,kf.
7. machine learn*.tw,kf.
8. deep learn*.tw,kf.
9. computer vision.tw,kf.
10. natural language process*.tw,kf.
11. 1 or 2 or 3 or 4 or 5 or 6 or 7 or 8 or 9 or 10
12. exp transitional cell carcinoma/
13. exp bladder cancer/
14. exp ureter cancer/
15. exp urethra cancer/
16. ((urothelial or transitional cell* or transition cell*) adj3 (carcinoma or neoplasm or cancer)).tw,kf.
17. (bladder adj3 (carcinoma or neoplasm or cancer)).tw,kf.
18. (ureteral adj3 (carcinoma or neoplasm or cancer)).tw,kf.
19. (urethral adj3 (carcinoma or neoplasm or cancer)).tw,kf.
20. ((renal pelvis or ureteropelvic junction) adj3 (carcinoma or neoplasm or cancer)).tw,kf.
21. 12 or 13 or 14 or 15 or 16 or 17 or 18 or 19 or 20
22. 11 and 21

Scopus
TITLE-ABS-KEY ("artificial intelligence" OR "artificial intelligent" OR "machine learning" OR "deep learning" OR "neural network" OR "computer vision" OR "computer-assisted diagnosis" OR "forecast" OR "natural language processing") AND TITLE-ABS-KEY ("urothelial cancer" OR "urothelial neoplasm" OR "urothelial carcinoma" OR "transitional cell carcinoma" OR "bladder cancer" OR "bladder neoplasm" OR "bladder carcinoma" OR "urethral cancer" OR "urethral neoplasm" OR "urethral carcinoma" OR "ureteral cancer" OR "ureteral neoplasm" OR "ureteral carcinoma" OR "renal pelvis cancer" OR "renal pelvis neoplasm" OR "renal pelvis carcinoma")

Web of Science
((artificial intelligen*) OR (machine learn*) OR (deep learn*) OR (neural network*) OR (computer vision) OR (computer-assisted diagnosis) OR (natural language process*)) AND ((urothelial cancer) OR (urothelial neoplasm) OR (urothelial carcinoma) OR (transition* cell carcinoma) OR (bladder cancer) OR (bladder neoplasm) OR (bladder carcinoma) OR (urethral cancer) OR (urethral neoplasm) OR (urethral carcinoma) OR (ureteral cancer) OR (ureteral neoplasm) OR (ureteral carcinoma) OR (renal pelvis cancer) OR (renal pelvis neoplasm) OR (renal pelvis carcinoma))

Supplementary Table 1. Interrater reliability of APPRAISE-AI item, domain, and overall scores determined by intraclass correlation coefficients (two-way random effects, absolute agreement, single measurement).

**Item**	**Interrater Reliability (95% CI)**
Title	1
Background	0.61 (0.16-0.85)
Objective and problem	1
Source of data	0.97 (0.92-0.99)
Eligibility criteria	0.64 (0.20-0.86)
Ground truth	0.67 (0.25-0.88)
Data abstraction, cleaning, preparation	0.88 (0.67-0.96)
Data splitting	0.95 (0.85-0.98)
Sample size calculation	1
Baseline	0.92 (0.79-0.97)
Model description	0.60 (0.13-0.85)
Hyperparameter tuning	0.78 (0.46-0.92)
Cohort characteristics	0.97 (0.91-0.99)
Model specification	0.73 (0.37-0.90)
Model evaluation	0.91 (0.75-0.97)
Clinical utility assessment	1
Bias assessment	0.77 (0.46-0.92)
Error analysis	1
Model explanation	0.62 (0.17-0.85)
Critical analysis	1
Implementation into clinical practice	1
Limitations	0.88 (0.68-0.96)
Disclosures	1
Transparency	0.96 (0.88-0.99)
**Domain**	**Interrater Reliability (95% CI)**
Clinical relevance	0.83 (0.57-0.94)
Data quality	0.93 (0.80-0.98)
Methodological conduct	0.95 (0.86-0.98)
Robustness of results	0.96 (0.87-0.98)
Reporting quality	0.91 (0.77-0.97)
Reproducibility	0.94 (0.84-0.98)
**Overall score**	**0.98 (0.96-0.99)**

Supplementary Figure 1. Temporal trend in overall APPRAISE-AI scores of the included studies.

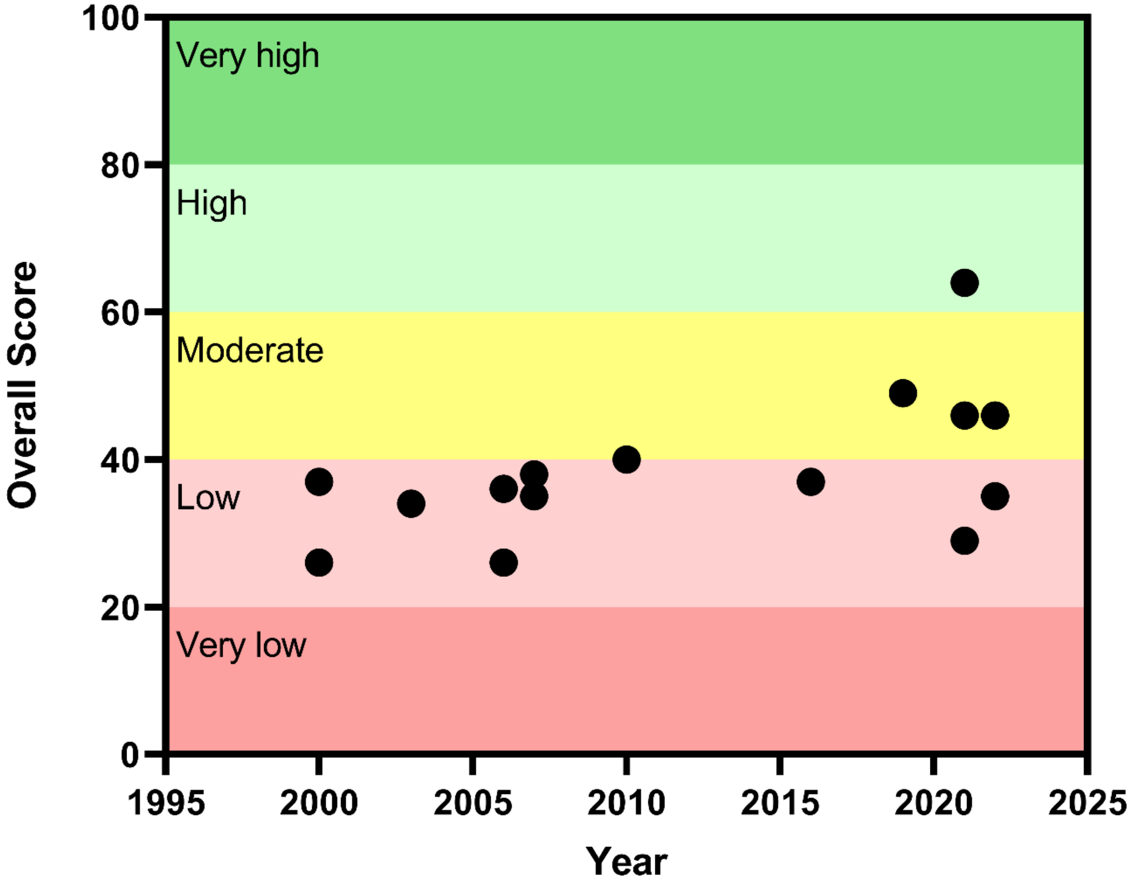

Supplementary Figure 2. Mean APPRAISE-AI item scores for the 15 studies using AI to predict NMIBC recurrence and progression. Each field is presented as a percentage of the maximum possible score for that field (i.e., mean score/maximum possible score x 100%) to compare scores between fields, irrespective of the assigned weighting. Items are coloured based on percentage of their corresponding maximum possible score: red for less than 40%, blue for between 40 and 60%, and green for greater than or equal to 60%.

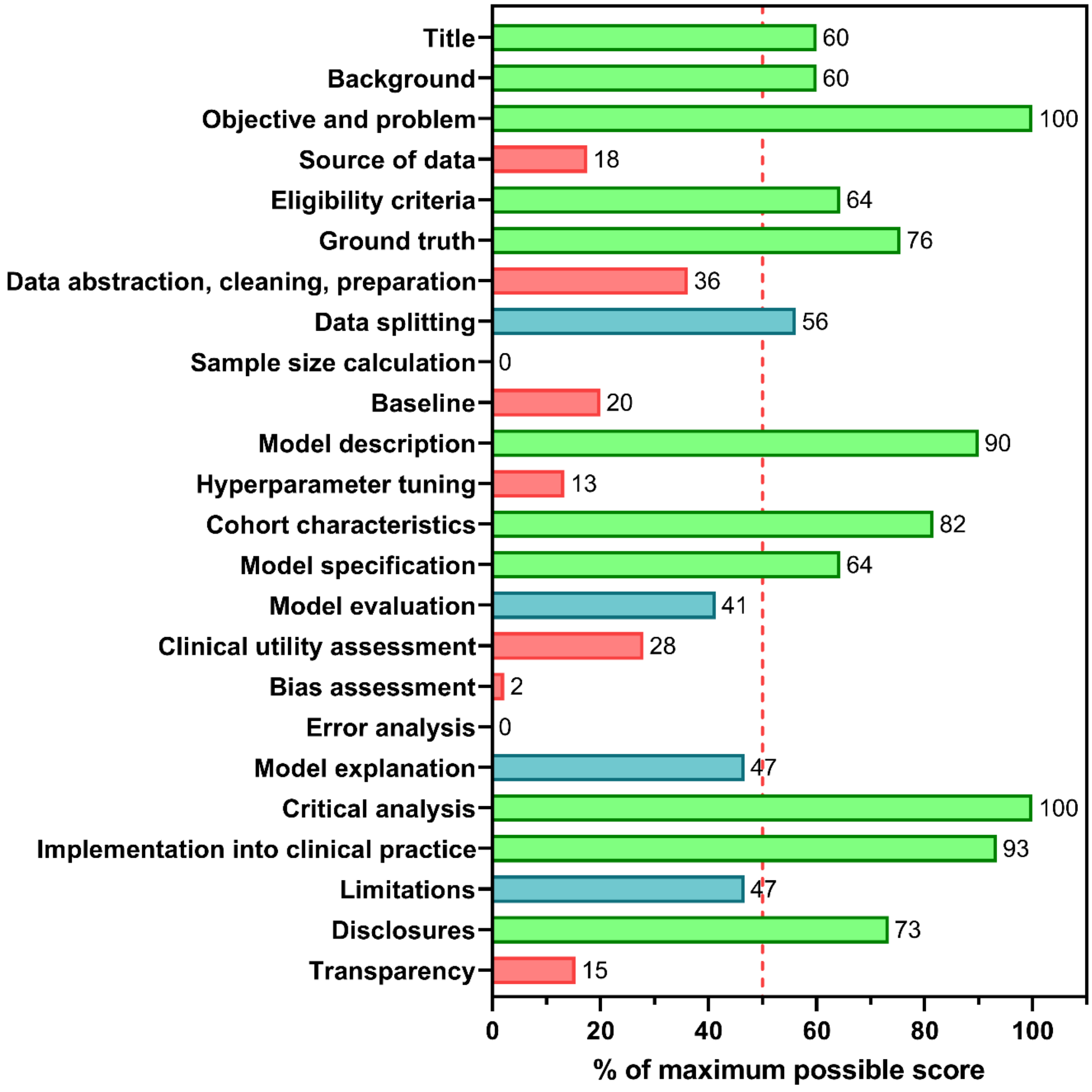

Supplementary Figure 3. Box plot of absolute differences in performance metrics (accuracy, c-index, sensitivity, and specificity) between AI and non-AI approaches, stratified by study quality according to overall APPRAISE-AI scores. Each box represents the 25th and 75th percentiles with the center line indicating the median, and the whiskers extending to the minimum and maximum differences. The number of studies in each group are indicated in parentheses.

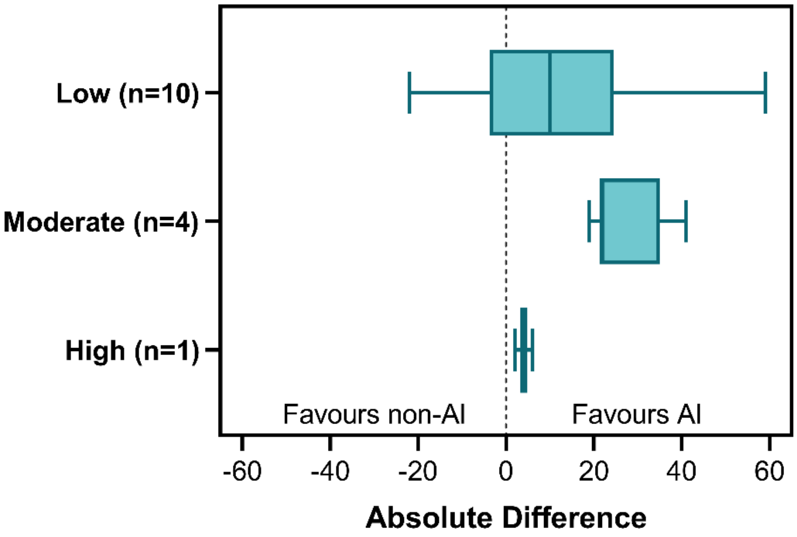

Supplementary Note 2. Sample size calculation. A sample size calculation is provided for a new prediction model with 10 features. Assumptions are based on the study cohort from the EAU Prognostic Risk Groups, which included 3401 patients with primary NMIBC who did not receive intravesical bacillus Calmette-Guérin.[1] Of these, 168 patients (5%) developed progression during a median follow-up of 3.9 years. The EAU risk groups achieved a bias-corrected c-index of 0.80 at 5 years. A shrinkage of 0.9 was used for sample size calculations, using R code from Riley et al.[2,3]

```
# Install required packages
install.packages("pmsampsize")
install.packages("rms")

# Load required packages
library("pmsampsize")
library("rms")

# Since, the Cox-Snell R-squared value is not reported in the study, this was determined
# using the following code by Riley et al (2021).

approximate_R2 = function(auc, prev, n = 1000000){

	# define mu as a function of the C statistic
	mu  = sqrt(2) * qnorm(auc)

	# simulate large sample linear prediction based on two normals
	# for non-eventsN(0, 1), events and N(mu, 1)
	LP = c(rnorm(prev*n,  mean=0, sd=1), rnorm((1-prev)*n, mean=mu, sd=1))
	y = c(rep(0, prev*n), rep(1, (1-prev)*n))

	# Fit a logistic regression with LP as covariate;
	# this is essentially a calibration model, and the intercept and
	# slope estimate will ensure the outcome proportion is accounted
	# for, without changing C statistic
	fit = lrm(y~LP)
	max_R2 = function(prev){
		1-(prev^prev*(1-prev)^(1-prev))^2
		}

	return(list(R2.nagelkerke = as.numeric(fit$stats['R2']),
		     R2.coxsnell = as.numeric(fit$stats['R2']) * max_R2(prev)))
	}

set.seed(1234)

# This function gives a Cox-Snell R-squared value of 0.06218884
approximate_R2(auc = 0.8, prev = 0.05, n=1000000)

# With the Cox-Snell R-squared value above, the following sample size
# was determined using the following code below by Riley et al (2019).

pmsampsize(type='s', rsquared=0.06218884, parameters=10, shrinkage=0.9,
		rate=0.05, timepoint=5, meanfup=3.9)

# The following output is provided:
# Minimum sample size required for new model development based on user
# inputs = 1397, corresponding to 5448.3 person-time** of follow-up, with
# 273 outcome events assuming an overall event rate = 0.05 and therefore
# an EPP = 27.24.
```

Supplementary References

1. Sylvester RJ, Rodríguez O, Hernández V, Turturica D, Bauerová L, Bruins HM, et al. European Association of Urology (EAU) Prognostic Factor Risk Groups for Non–muscle-invasive Bladder Cancer (NMIBC) Incorporating the WHO 2004/2016 and WHO 1973 Classification Systems for Grade: An Update from the EAU NMIBC Guidelines Panel. European Urology. 2021 Apr 1;79(4):480–8.

2. Riley RD, Van Calster B, Collins GS. A note on estimating the Cox-Snell R2 from a reported C statistic (AUROC) to inform sample size calculations for developing a prediction model with a binary outcome. Statistics in Medicine. 2021;40(4):859–64.

3. Riley RD, Snell KI, Ensor J, Burke DL, Harrell FE, Moons KG, et al. Minimum sample size for developing a multivariable prediction model: PART II - binary and time-to-event outcomes. Stat Med. 2019 Mar 30;38(7):1276–96.
